# Coffee with High but Not Low Caffeine Content Augments Fluid and Electrolyte Excretion at Rest

**DOI:** 10.3389/fnut.2017.00040

**Published:** 2017-08-18

**Authors:** Adam D. Seal, Costas N. Bardis, Anna Gavrieli, Petros Grigorakis, J. D. Adams, Giannis Arnaoutis, Mary Yannakoulia, Stavros A. Kavouras

**Affiliations:** ^1^Hydration Science Laboratory, University of Arkansas, Fayetteville, AR, United States; ^2^Department of Nutrition and Dietetics, Harokopio University, Athens, Greece; ^3^Division of Endocrinology, University of Arkansas for Medical Science, Little Rock, AR, United States

**Keywords:** caffeine, fluid balance, electrolytes, hydration, coffee, dehydration, hypohydration

## Abstract

**Background:**

Low levels of caffeine ingestion do not induce dehydration at rest, while it is not clear if larger doses do have an acute diuretic effect. The aim of the present investigation was to examine the acute effect of low and high levels of caffeine, *via* coffee, on fluid balance in habitual coffee drinkers (at least one per day) at rest.

**Methods:**

Ten healthy adults (eight males and two females; age: 27 ± 5 years, weight: 89.5 ± 14.8 kg, height: 1.75 ± 0.08 m, and body mass index: 29.1 ± 4.4 kg m^−2^) ingested 200 mL of water (W), coffee with low caffeine (3 mg kg^−1^, LCAF), or coffee with high caffeine (6 mg kg^−1^, HCAF) on three respective separate occasions. All sessions were performed at 09:00 in the morning in a counterbalanced, crossover manner, at least 5 days apart. Subjects remained in the laboratory while urine samples were collected every 60 min for 3 h post ingestion.

**Results:**

Absolute caffeine consumption was 269 ± 45 and 537 ± 89 mg for the LCAF and HCAF, respectively. Coffee ingestion at the HCAF trial induced greater diuresis during the 3-h period (613 ± 101 mL, *P* < 0.05), when compared to W (356 ± 53 mL) and LCAF (316 ± 38 mL). In addition, cumulative urinary osmotic excretion was significantly greater in the HCAF (425 ± 92 mmol, *P* < 0.05), as compared to the W (249 ± 36 mmol) and LCAF (177 ± 16 mmol) trials.

**Conclusion:**

The data indicate that caffeine intake of 6 mg kg^−1^ in the form of coffee can induce an acute diuretic effect, while 3 mg kg^−1^ do not disturb fluid balance in healthy casual coffee drinking adults at rest.

## Introduction

Maintaining fluid balance is vital for health and well-being (1, 2). Fluid balance can be compromised by failing to consume sufficient fluid to meet water losses, leading to hypohydration. Although there are national guidelines on total water intake (including fluids and food) (3), there is no clear consensus on the effectiveness of different fluids on hydration. The US department of agriculture in the 2015–2020 dietary guidelines for Americans suggested to consume “beverages with no added sugars, such as water, in place of sugar-sweetened beverages,” in an effort to decrease the added sugar intake (4). The guidelines for consuming caffeinated beverages, such as coffee, and their effect on body fluid balance are less clear. The United States Food and Drug Administration recommends no more than 400 mg day^−1^ of caffeine in healthy adults equating to approximately three to four cups of coffee. However, this is a standard amount not relative to body weight, which has been shown to effect caffeine metabolism (5). Moreover, the recommendation fails to declare an amount capable of disrupting fluid balance (6). In 2015, the European Food Safety Authority concluded caffeine ingestion up to 6 mg kg^−1^ of body weight will not induce diuresis; however, their conclusion only pertained to caffeine ingestion coupled with endurance exercise (7).

Coffee is a pharmacologically active, socially accepted, and widely consumed beverage. Over the past decade, scientific interest in relation to coffee has increased as new light is shed on coffee’s potential health benefits (8). Habitual coffee intake has been associated with reduced risk of type 2 diabetes (9), metabolic syndrome (10), and obesity (11). Habitual coffee consumption has been inversely associated with inflammatory markers in healthy and/or diabetic adults (12). In addition, in a cohort of over 400,000 men and women, coffee ingestion was inversely associated with observed deaths due to heart disease, respiratory disease, stroke, diabetes, and infections (13).

Caffeine (1,3,7-trimethylxanthine), a naturally occurring methylxanthine also found in tea, chocolate, and energy drinks, acts as a competitive adenosine receptor antagonist to reduce fractional sodium reabsorption in both the proximal tubule and distal nephron (14, 15). Due to this phenomenon, there have been a range of studies and reviews investigating the effects of caffeine on fluid balance during exercise (16, 17) and at rest (18, 19). While the data vary, it seems that higher doses of caffeine will acutely increase urinary output, while lower to moderate doses of caffeine will not have a diuretic effect.

However, few studies have specifically investigated the effects of caffeine in the form of coffee on fluid balance and electrolyte excretion. This is surprising considering coffee holds second position in consumption among all beverages after water (20). One study conducted by Neuhauser et al. investigated the effects of a high volume of coffee (624 mg caffeine) on urine excretion following a 5-day caffeine deprivation period (21). Such a high dose of caffeine caused a 2.7% decrease in total body water and a 41% increase in urine excretion, with a subsequent 66 and 28% increase in urinary sodium and potassium excretion over a 24-h period. However, the dose provided was a standard amount not relative to bodyweight. Killer et al. examined the effect of caffeine against water intake in 50 regular coffee drinkers who consume 3–6 cups of coffee. They reported no increases in urine volume (UV), specific gravity, or osmolality during a 24-h period following coffee ingestion with 4 mg kg^−1^ caffeine in the form of coffee (range of 204–453 total mg, average dose of 308 mg). However, there was a slight increase in urinary sodium excretion (22). Although this study utilized labeled isotope D_2_O to track total body water and a dose of caffeine relative to bodyweight, only one dose (4 mg kg^−1^) was investigated.

To the authors’ knowledge, these are the only two studies directly investigating the diuretic effects of caffeinated coffee. The studies utilized different doses of caffeine (624 and 308 mg, respectively) and reached different conclusions regarding diuresis. Consequently, it is important to elucidate fluid balance characteristics of coffee and to observe its dose–responses on hydration markers. The aim of this study was to compare the acute effects of low and high caffeinated coffee ingestion on fluid balance in habitual coffee drinkers at rest.

## Materials and Methods

### Participants

Ten healthy adults (eight males and two females; age: 27 ± 5 years, weight: 89.5 ± 14.8 kg, height: 1.75 ± 0.08 m, and body mass index: 29.1 ± 4.4 kg m^−2^) were recruited by local advertisement to participate in this study. Exclusionary criteria included smoking, specialized diets, participation in competitive sports, as well as hypertension, metabolic disease, gastrointestinal conditions, or recent surgical incidents. All participants were habitual coffee drinkers (1–3 cups per day). The protocol was approved by the Harokopio University Ethics Committee. Subjects provided informed written consent in accordance with the Declaration of Helsinki.

### Study Design

Study participants completed three trials on separate occasions at least 5 days apart in a counterbalanced, crossover manner. All sessions took place in the morning at 09:00 a.m. Participants refrained from ingesting foods or drinks with caffeine or other methylxanthines 24 h before each trial. After an overnight fast, they arrived at the laboratory, provided a urine sample and emptied their bladders. They consumed a standard breakfast composed of one slice of white bread with 5 g of butter and 10 g of sugar, within 5 min. Then they ingested 200 mL of water, 200 mL of instant coffee with low caffeine (3 mg kg^−1^, LCAF), or 200 mL of instant coffee with high caffeine (6 mg kg^−1^, HCAF). The HCAF and LCAF trials were performed in a single blind fashion. The amount of fluid consumed during each trial did not change; however, the amount of coffee and concentration of caffeine in the 200 mL of fluid differed between treatments. This process was to ensure similar fluid balance at the onset of each trial. After ingesting coffee or water, volunteers rested in seating position in the laboratory for 3 h to allow for collection of urine samples.

### Urine Sample Analysis

Urine was collected at 60, 120, and 180 min after the test drink ingestion and UV was measured. If a volunteer needed to void outside of this structured time frame, this volume was added to the cumulative volume during the corresponding hour. The samples were analyzed fresh and in duplicate for osmolality and electrolyte content. Urine osmolality (UOsm) was measured *via* freezing point depression (3D3 osmometer, Advanced Instruments Inc., Norwood, MA, USA), while potassium and sodium *via* an electrolyte sensitive analyzer (Ilyte Na/K/Li Analyzer; Instrumentation Laboratory, Milan, Italy). Urinary osmotic or electrolyte excretion was determined by multiplying the volume and concentration of samples for each time-point. Cumulative values were also reported to present the overall effect over the 3-h timeline.

### Statistical Analysis

A two-way repeated measures ANOVA with Bonferroni correction was used to detect differences between trials. All statistical procedures were completed using JMP Pro 13. Data are presented as means ± SD of the means unless stated otherwise. Significance was set at the *P* < 0.05 level.

## Results

Volunteers consumed 269 ± 45 and 537 ± 89 mg of caffeine for LCAF and HCAF trials, respectively, representing a low and high dose of caffeine.

### Urinary Analysis

**Figure 1:**
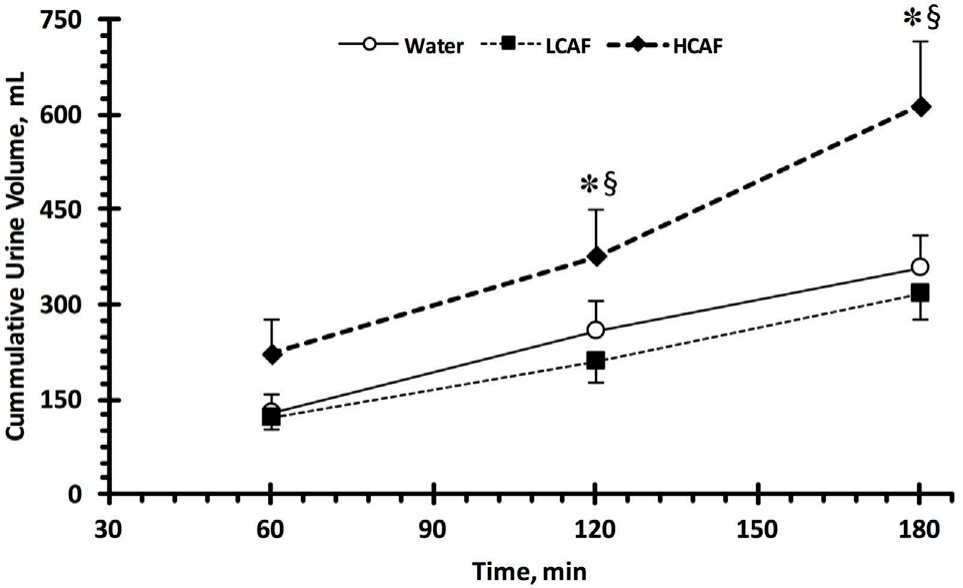
Cumulative urinary output during the three trials. Low caffeine (LCAF) intake (3 mg kg^−1^); high caffeine (HCAF) intake (6 mg kg^−1^). Error bars depict SEM. * denotes statistically significant different from water trial during the same time-point. § denotes statistically significant different from LCAF trial during the same time-point.

**Figure 2:**
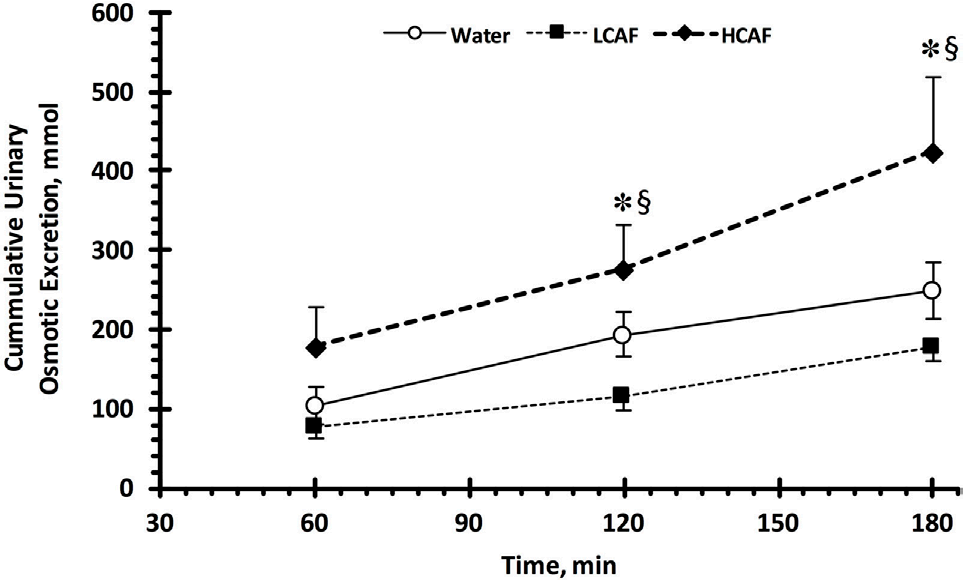
Cumulative urinary osmotic excretion. Low caffeine (LCAF) intake (3 mg kg^−1^); high caffeine (HCAF) intake (6 mg kg^−1^). Error bars depict SEM. * denotes statistically significant different from water trial during the same time-point. § denotes statistically significant different from LCAF trial during the same time-point.

Table 1 contains the urinary analysis results at each time-point (60, 120, and 180 min) and treatment (W, LCAF, and HCAF) during the 3-h period after the 200-mL bolus of either water or the two coffee trials. HCAF trial resulted in higher urinary output during most time-points when compared to LCAF and W trials (*P* < 0.05). The cumulative UV (mL) during the 3-h period after the 200-mL bolus of either water or the two coffee trials is presented in Figure 1. Cumulative urinary output was significantly greater in the HCAF trial (*P* < 0.05) when compared to LCAF and the W trial at the 2- and 3-h mark (Figure 1); no differences existed between W and LCAF at any time-points (*P* > 0.05). UOsm was not different between treatments or time-points among trials (*P* < 0.05). However, cumulative urinary osmotic excretion was significantly higher during the HCAF trial than the LCAF and W trial at the 2- and 3-h time-points, without any differences between LCAF and W (*P* < 0.05, Figure 2). Urine sodium concentration was significantly higher at all three time-points of HCAF as compared to the corresponding time-points of the water trial and the 120- and 180-min time-point of the LCAF trial (*P* < 0.05). Cumulative urinary sodium excretion at the 180-min time-point was significantly higher in HCAF as compared to the same time-point of water and LCAF (*P* < 0.05). Cumulative urinary potassium excretion was significantly higher at the 180-min time-point of HCAF as compared to the W and LCAF trial.

**Table 1:** UV and electrolyte excretion during the water (W), LCAF (3 mg kg^−1^), and HCAF (6 mg kg^−1^) trials.

Time, min	W				LCAF (3 mg kg^−1^)				HCAF (6 mg kg^−1^)			
	0	60	120	180	0	60	120	180	0	60	120	180
UV, mL	–	128 ± 92	128 ± 100	101 ± 65	–	121 ± 64	88 ± 59	107 ± 56	–	221 ± 178^b^	153 ± 145^b^	239 ± 157^a^^,^^b^
UOsm, mmol kg^−1^	867 ± 164	788 ± 152	620 ± 96	612 ± 68	790 ± 101	746 ± 279	560 ± 196	636 ± 187	898 ± 182	759 ± 194	609 ± 213	728 ± 103
UOsm exc, mmol	–	103 ± 68	91 ± 64	55 ± 40	–	77 ± 42	39 ± 23	62 ± 38	–	179 ± 138^b^	96 ± 94	149 ± 106^a^^,^^b^
U[Na^+^], mmol/L	63.4 ± 19.3	58.1 ± 7.7	68.4 ± 10.5	81.0 ± 14.0	79.2 ± 37.7	80.9 ± 17.0	66.9 ± 11.8	70.6 ± 34.0	93.1 ± 12.7	98.0 ± 14.2^a^	92.8 ± 19.3^a^^,^^b^	109.1 ± 27.9^a^^,^^b^
U[K^+^], mmol/L	35.2 ± 15.0	35.6 ± 10.9	39.5 ± 11.3	51.2 ± 16.2	41.8 ± 10.9	36.8 ± 6.6	45.1 ± 7.4	46.3 ± 4.3	34.7 ± 4.2	37.4 ± 4.6	44.0 ± 9.8	50.6 ± 7.3
UNa^+^ exc, mmol	–	8.1 ± 5.2	11.2 ± 8.0	6.9 ± 5.4	–	9.0 ± 6.2	5.0 ± 3.7	6.8 ± 5.0	–	19.4 ± 20.3^a^	11.3 ± 7.7	18.6 ± 13.3^a^^,^^b^
UK^+^ exc, mmol	–	4.9 ± 3.7	6.5 ± 5.0	4.8 ± 4.0	–	4.2 ± 3.0	3.6 ± 3.2	4.5 ± 2.8	–	8.8 ± 7.2	4.9 ± 3.1	8.5 ± 5.7
Cum. UNa^+^ exc, mmol	–	8.1 ± 5.2	19.3 ± 9.1	26.2 ± 10.6	–	9.0 ± 6.2	14.0 ± 8.2	20.7 ± 8.7	–	19.4 ± 20.3	30.7 ± 17.8	49.3 ± 30.5^a^^,^^b^
Cum. UK^+^ exc, mmol	–	4.9 ± 3.7	11.4 ± 6.3	16.2 ± 8.7	–	4.2 ± 3.0	7.8 ± 5.1	12.2 ± 5.0	–	8.8 ± 7.2	13.8 ± 6.0	22.2 ± 11.4^b^

*^a^Denotes statistically significant different from Water trial during the same time-point*.

*^b^Denotes statistically significant different from LCAF trial during the same time-point*.

*UV, urine volume; UOsm, urine osmolality; U[Na^+^], urine sodium concentration; U[K^+^], urine potassium concentration; exc, excretion; LCAF, low caffeine; HCAF, high caffeine*.

## Discussion

The main finding of this study was that ingestion of highly caffeinated coffee (6 mg kg^−1^) resulted in an acute diuretic effect represented by higher cumulative UV 2 and 3 h after ingestion. UV and cumulative urinary osmotic excretion were higher 3-h after ingestion of 6 mg kg^−1^ of caffeine as compared to 3 mg kg^−1^ or water. This finding provides insight into the specific amount of caffeine relative to bodyweight and in the form of coffee that elicits a diuretic response. Participants during the HCAF treatment averaged 537 mg as compared to 269 mg of caffeine consumption in the LCAF treatment. A standard cup of coffee contains 95–165 mg of caffeine (23). Therefore, daily consumers who ingest approximately two to three cups of coffee will likely not experience significant disruptions in fluid balance. However, habitual coffee drinkers who approach approximately four or more cups per day could experience caffeine-induced diuresis. Caffeine levels in coffee do, however, vary depending on where and when a consumer purchases the coffee. A study by McCusker et al. analyzed disparities in average caffeine levels between popular coffee vendors. The range of average caffeine across all vendors was 58–259 mg per dose, which remains under the diuretic threshold reported in this study. The researchers also analyzed the day-to-day variations in caffeine levels of a single vendor reporting range of 259–564 mg/dose. Interestingly, on Monday and Tuesday, the average caffeine content was 564 and 498 mg, respectively, as compared to the rest of the week in which caffeine levels were approximately 300 mg (24). Considering these data, consumers of commercial coffee could encounter caffeine levels comparable to those investigated in the HCAF treatment of this study.

The dose-dependent pattern of diuresis remains evident in experiments during which participants consumed caffeine from sources other than coffee. Several studies have reported caffeine-induced diuresis at doses greater than 500 mg (21, 25, 26); however, in studies administering lower doses, diuresis seems not to exist. Armstrong et al. conducted an 11-day experiment in which participants ingested either 0, 226, or 452 mg of pure caffeine in tablet form. There were no reported differences in UV regardless of higher dosages (19). Zhang et al. performed a meta-analysis utilizing 28 investigations (mean dose = 300 mg) with change in UV as the main outcome measure. In this analysis, the overall effect size for caffeine-induced diuresis was small (0.29); however, when studies including exercise were omitted, the effect size increased to moderate (0.54) (27). This finding indicates that exercise may reduce the diuretic effect of caffeine. Exercise activates sympathoadrenal action, thereby releasing catecholamines and causing renal arteriole constriction, thus lowering glomerular filtration rate and offsetting caffeine-induced diuresis (15, 28, 29). This phenomenon has been shown in a previous study, during which increased levels of catecholamines were reported (25). Maughan and Griffin conducted a review of 11 studies examining caffeine-induced diuresis. The researchers concluded doses of caffeine >250 mg elicited diuretic effects while smaller doses did not. These conflicting results at approximately 300 mg may be explained by inter-individual variability stemming from factors affecting caffeine metabolism, such as genetics, physical activity, bodyweight, gender, and nutritional status (5, 27, 30). However, the trend of diuresis occurring at doses of approximately 500 mg is still supported, as seen in this study.

Cumulative urinary osmotic excretion was higher in HCAF trials as compared to LCAF trials. Although there was a slight increase in potassium excretion, this finding is probably due to increased sodium excretion in HCAF trials. These results are in line with Killer et al., who reported higher urinary sodium excretion following ingestion of 4 mg kg^−1^ of caffeine in the form of coffee (22). This increase in sodium excretion also falls in line with previous studies utilizing caffeine sources other than coffee (31, 32). It has been suggested that this effect may be a result of decreased sodium reabsorption in the proximal tubules (22). As more sodium is excreted through urine, water will also be excreted. Natriuresis was also reported with other methylxanthines, such as aminophylline and theophylline, which act similarly to reduce sodium reabsorption (33). This may be a mechanism for the increased UV and sodium concentration seen in the HCAF trial of this study.

This study has several strengths and limitations. Most of the studies investigating the diuretic effects of caffeine were conducted with pure caffeine tablets or sources other than coffee. The use of isolated caffeine reduces potential variability in results considering the presence of other bioactive components in coffee; however, coffee is the second most consumed beverage in the world after water. Therefore, the use of coffee as the method for caffeine delivery could be considered among the strengths of this study and makes the results generalizable to larger populations. In addition, several previous studies administered a standard dose of caffeine not relative to bodyweight. As explained by Kamimori et al., bodyweight may influence the pharmacokinetics of caffeine metabolism (5) and should, therefore, be controlled as in this study. Among the limitations is the small sample size; in addition, the two genders are not equally represented. Zhang et al. reported sixfold higher caffeine-induced diuresis in women as compared to men (27). Perhaps the diuretic effect would have been more pronounced with a balanced sample of genders. Also, urine was only collected for 3-h post ingestion. The plasma half-life of caffeine can range from 2.3 to 12 h (34). Although the specific purpose was to examine the acute response, it may have been interesting to utilize 24-h collection to examine the total time frame in which coffee-induced diuresis remained. Due to time constraints, this was not possible. Lastly, we should mention that our finding might apply only to habitual coffee drinkers. The effects of a single cup of coffee with 6 mg kg^−1^ will likely be different if consumed by a naive coffee drinker as opposed to a habitual coffee drinker as in this study.

Having considered study’s limitations, the results suggest caffeinated coffee in higher doses (6 mg kg^−1^) induces an acute diuretic effect. This effect is not seen with lower doses (3 mg kg^−1^) or plain water. Therefore, habitual consumers of coffee not exceeding 3 mg kg^−1^ of caffeine should not worry about detrimental diuretic effects. However, once consumers approach 6 mg kg^−1^ of caffeine potential disruptions in fluid balance should be considered.

## Ethics Statement

The protocol was approved by the Harokopio University Ethics Committee. Subjects provided informed written consent in accordance with the Declaration of Helsinki.

## Author Contributions

SK, MY, and AG designed research; CB, AG, PG, and GA, conducted data collection and sample analysis; AS, JA, CB, and SK analyzed the data; AS, JA, and SK wrote the paper. SK was the principal investigator and had primary responsibility for the final content. All authors read, critically revised, and approved the final manuscript.

## Conflict of Interest Statement

SK was a scientific consultant for Quest Diagnostic and has active grants with Danone Research. AS is a scientific consultant for Gatorade Sports Science Institute. CB, AG, JA, PG, GA, and MY have no conflict to declare.
